# An AAAG-Rich Oligodeoxynucleotide Rescues Mice from Bacterial Septic Peritonitis by Interfering Interferon Regulatory Factor 5

**DOI:** 10.3390/ijms18051034

**Published:** 2017-05-11

**Authors:** Shuang Gao, Xin Li, Shu Nie, Lei Yang, Liqun Tu, Boqi Dong, Peiyan Zhao, Yangyang Wang, Yongli Yu, Liying Wang, Shucheng Hua

**Affiliations:** 1 Department of Molecular Biology in College of Basic Medical Sciences and Institute of Pediatrics in First Hospital, Jilin University, Changchun 130021, China; shuangg@jlu.edu.cn (S.G.); xliin@126.com (X.L.); nieshu51@163.com (S.N.); yangleistudent@163.com (L.Y.); paris_518@sina.com (P.Z.); wyystudent@163.com (Y.W.); 2 Department of Immunology, College of Basic Medical Sciences, Jilin University, Changchun 130021, China; tulq15@mails.jlu.edu.cn (L.T.); dongbq14@mails.jlu.edu.cn (B.D.); ylyu@jlu.edu.cn (Y.Y.); 3 Department of Endodontics, Jilin Provincial Key Laboratory of Tooth Development and Bone Remodeling, School and Hospital of Stomatology, Jilin University, Changchun 130021, China; 4 Department of Respiratory Medicine, The First Hospital of Jilin University, Changchun 130021, China

**Keywords:** septic peritonitis, oligodeoxynucleotide, interferon regulatory factor 5, inflammation, macrophage

## Abstract

A previous study found that an AAAG-rich Oligodeoxynucleotide (ODN), designated as MS19, could lessen the acute lung inflammatory injury (ALII) in mice infected by influenza viruses. Bioinformatics analysis found that MS19 is consensus with the binding site of interferon regulatory factor 5 (IRF5) in the regulatory elements of pro-inflammatory genes. This study established a septic peritonitis model in Institute of Cancer Research (ICR) mice infected with *Escherichia coli* (*E. coli*), and found that MS19 prolonged the survival of the mice and down-regulated the expression of inducible nitric oxide synthase (iNOS), interleukin-6 (IL-6), and tumor necrosis factor α (TNF-α). In cultured RAW264.7 cells, MS19 significantly reduced the expression of iNOS, IRF5, IL-6, and TNF-α and inhibited the nuclear translocation of IRF5. This data may provide a new insight for understanding how MS19 reduces the excessive inflammatory responses in sepsis.

## 1. Introduction

The septic peritonitis is the host’s systemic inflammatory response to the bacteria, initiated by pathogen associated molecule patterns (PAMPs) including lipopolysaccharide (LPS) and lipid A from γ-negative bacteria and lipoteichoic acid (LTA) and peptidoglycan from γ-positive bacteria [1]. Most sepsis is caused by bacteria, and bacteria of abdominal origin contribute to the second major reason for sepsis. Sepsis is defined as life-threatening organ dysfunction caused by a dysregulated host response to infection [2]. The prevalence of sepsis internationally is estimated at 300 per 100,000 [3]. Sepsis is now recognized to be involved in the early activation of both pro- and anti-inflammatory responses [4], along with major modifications in non-immunologic pathways including cardiovascular, neuronal, autonomic, hormonal, bioenergetic, metabolic, and coagulation pathways, all of which have prognostic significance [5].

The pathogenesis of sepsis includes countless disturbances of the host immune system starting with a harmful, infection-triggered exaggerated inflammatory cascade [6]. Numerous exogenous (endo- and exotoxin) and endogenous mediators are capable of activating monocytes and macrophages, which synthesize cytokines [6]. In this process, macrophages produce a great amount of pro-inflammatory cytokines that lead to host mortality [7,8]. Therefore, the macrophages could be a potential target for preventing infection-triggered exaggerated inflammation [8]. Functionally, macrophages are classified into M1 and M2 categories [9]. M1 macrophages are classic or pro-inflammatory macrophages that produce tumor necrosis factor α (TNF-α), interleukin-1 (IL-1), interleukin-6 (IL-6), and inducible nitric oxide synthase (iNOS). M1 macrophages have strong microbial activity, promote inflammation, and eliminate pathogens. M2 macrophages are alternatively activated macrophages that produce IL-10, Arginase-1, and Mrc1 (CD206). M2 macrophages are involved in preventing excessive injury in host tissue remodeling. M1 macrophages and M2 macrophages are “heal” type macrophages that routinely repair and maintain tissue integrity. The imbalance of M1/M2-type response causes diseases. In sepsis, inflammatory response (M1-type) is very important for eliminating pathogens at earlier stages of infections [10]. However, once macrophages are activated, the release of TNF and other mediators are implicated in the pathogenesis of acute septic shock [5].

Interferon regulatory factor 5 (IRF5) is a master transcription factor that is generally involved downstream of the toll-like receptor–myeloid differentiation primary response gene 88 (TLR–MyD88) signaling pathway in the activation of genes for inflammatory cytokines [11]. The IRF5 was reported as a reliable marker for macrophages at sites of inflammation. IRF5 expression is induced during differentiation of human monocytes and murine bone marrow–derived macrophages into M1 macrophages [12,13]. In turn, IRF5 directly induces the production of pro-inflammatory cytokines such as IL-6 and TNF-α [11,13]. Structurally, a nuclear localization signal (NLS) exists in both the DNA binding domain (DBD) and the interactive domain (IAD) of IRF5, respectively [14]. In the NLS, phosphorylation sites exist and can be phosphorylated. In a static image, latent IRF5 appears in the cytoplasm. Upon activation, IRF5 is phosphorylated and thereby translocated to the nucleus where it binds to the regulatory regions of its target genes [15]. The amino-terminal DBD of IRF5 is involved in recognition of target DNA sequences, with the putative binding motif (G/C)(A)AAA(N)_2-3_AAA (G/C)(T/C) [16]. The motif is in consensus with MS19, a microsatellite DNA mimicking oligodeoxynucleotide (MS ODN) [17] with a sequence of 5′-AAAGAAAGAAAGAAAGAAAGAAAG-3′ [18]. Alternatively, MS19 was also designated as AAAG-rich ODN to specify its sequence characteristics.

In previous work [18], MS19 was found to decrease the mortality of mice infected with influenza viruses by reducing acute lung inflammatory injury (ALII). The reduction was correlated with the reduced production of pro-inflammatory cytokines like TNF-α and significantly reduced infiltration of inflammatory cells like neutrophils in lung tissues. Subsequently, MS19 almost completely prohibited the development of intra-alveolar edema, consolidation, and profuse hemorrhages in the lungs. Upon the work and the consensus between the IRF5 binding site and MS19, it was hypothesized that MS19 could prevent the IRF5 from binding the regulatory elements of the pro-inflammatory gene and therefore inhibit the development of sepsis.

To find the underlying mechanisms on how MS19 reduces fatal inflammatory responses, a septic peritonitis model in Institute of Cancer Research (ICR) mice infected with *Escherichia coli* (*E. coli*) was established. By using the mouse model, it was found that MS19 can prolong the survival in model mice, down-regulate the expression of iNOS and IRF5, inhibit the polarization of M1 macrophages, interfere nuclear transportation of IRF5, and prevent the excessive activation of downstream inflammatory signals. This data may provide an insight into the mechanisms on how MS19 reduces the excessive inflammatory responses.

## 2. Results

### 2.1. MS19 Prolongs the Survival Time of Escherichia coli (E. coli) Infected Mice

**Figure 1:**
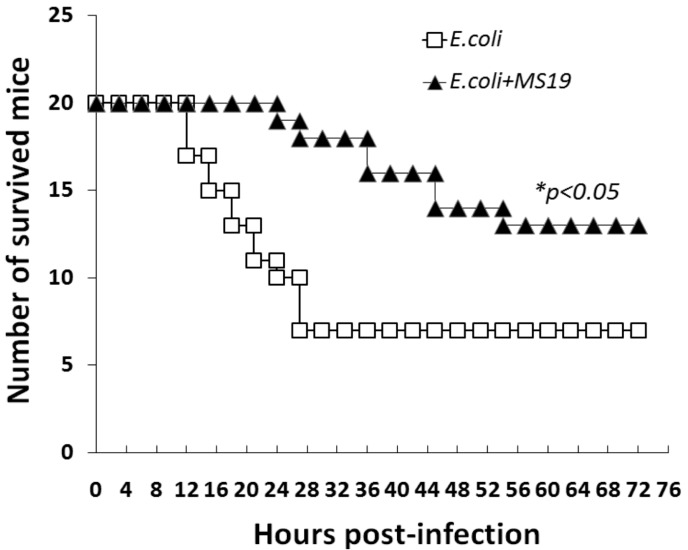
Survival curves of mice with septic peritonitis induced by *Escherichia coli* (*E. coli*.) 40 specific pathogen-free female Institute of Cancer Research (ICR) mice (20 ± 2 g) of eight-week-old were intraperitoneally injected with *E. coli* at 1.6 × 10^8^ Colony-Forming Units (CFUs). At 1 h post infection, the mice were intraperitoneally administrated with (*E. coli* + MS19, *n* = 20) or without (*E. coli*, *n* = 20) MS19 (10 µg/mouse) once. The mice were observed for 72 h for recording their survivals. * *p* < 0.05.

Considering that MS19 can reduce the ALII caused by influenza virus [18], and the excessive inflammatory response is the cause of the death of sepsis [5], this study tested whether MS19 was therapeutic to sepsis in mice. First, a mouse model of septic peritonitis was established. The mice were intraperitoneally injected with *E. coli* at 1.6 × 10^8^ Colony-Forming Units (CFUs) [19]. The infected mice started to die at 12 h post-*E. coli* injection. At 24 h after infection, 50% of the mice were dead. The survival rate of the mice was 35%. Comparatively, after being treated with MS19 at 1 h post-*E. coli* infection by intraperitoneal injection, all the mice survived 24 h post-infection. The survival rate of the mice treated by MS19 was 65% (*p* < 0.05) (Figure 1). The result suggested that MS19 could rescue mice from bacterial septic peritonitis.

### 2.2. MS19 Can Reduce the Production of Interleukin-6 (IL-6) and Tumor Necrosis Factor α (TNF-α) at 8 h after Infection

**Figure 2:**
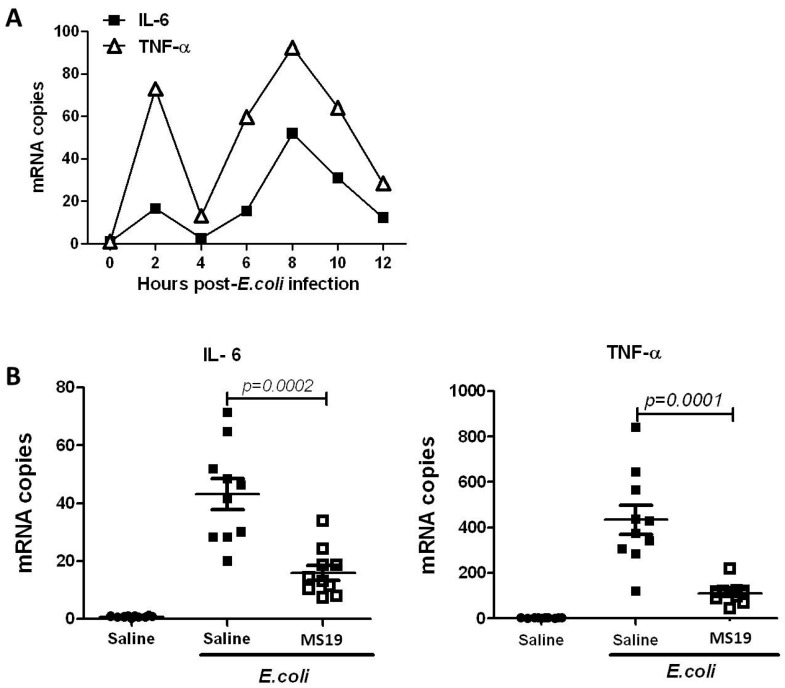
Effects of MS19 on the mRNA expression of IL-6 and TNF-α in peritoneal lavage cells (PLCs) of mice infected by *E. coli*. The mice were intraperitoneally injected with *E. coli* at 1.6 × 10^8^ CFUs. At 1 h post infection, the mice were intraperitoneally administrated with saline (A, *n* = 3) or with saline + MS19 (B, *n* = 10). The PLCs were harvested from the mice administrated with saline for 2 to 12 h. The PLCs were harvested from the mice administrated with saline + MS19 at 8 h post-*E. coli* infection. The PLCs were lysed to isolate total RNA for amplifying mRNA of IL-6 and TNF-α by quantitative reverse transcription-polymerase chain reaction (qRT-PCR). (**A**) Kinetic expression levels of IL-6 and TNF-α in PLCs of *E. coli*-infected mice without MS19 treatment; (**B**) Effects of MS19 on the mRNA expression levels of IL-6 and TNF-α in PLCs of *E. coli*-infected mice. Each point represents the mRNA level from one mouse. Mean ± standard deviation (SD) was marked with bars. The data were analyzed by SPSS 19.0 (San Francisco, CA, USA) and followed the Gaussian distribution.

This study tried to observe whether MS19 could inhibit the excessive inflammation induced by *E. coli*. Since the lipopolysaccharide (LPS) from the cell wall of γ-negative bacteria like *E. coli* causes sepsis by inducing pro-inflammatory mediators including TNF-α and IL-6 [20], this study detected whether MS19 could reduce the production of these inflammatory cytokines. First, peritoneal lavage cells (PLCs) were collected at different time points to observe the changes of inflammatory factors, and their mRNA expression of IL-6 and TNF-α were detected. As shown in Figure 2A, the mRNA levels of IL-6 and TNF-α in PLCs appeared at two peaks: The first was at 2 h post-*E. coli* infection and the second was at 8 h post-*E. coli* infection. Comparatively, the second peak was higher than the first: The IL-6 mRNA level was higher than the TNF-α mRNA level (Figure 2A). In parallel, the mRNA expression of IL-6 and TNF-α was detected in the PLCs collected from the mice infected with *E. coli* and treated with MS19 once at 1 h after *E. coli* infection. The PLCs were collected at 8 h post infection with *E. coli.* The result showed that MS19 significantly reduced the mRNA expression of IL-6 and TNF-α (Figure 2B), indicating that MS19 could alleviate the inflammatory response by inhibiting the expression of pro-inflammatory cytokines.

### 2.3. MS19 Down-Regulates Nitric Oxide Synthase (iNOS) Expression in the Peritoneal Lavage Cells (PLCs)

**Figure 3:**
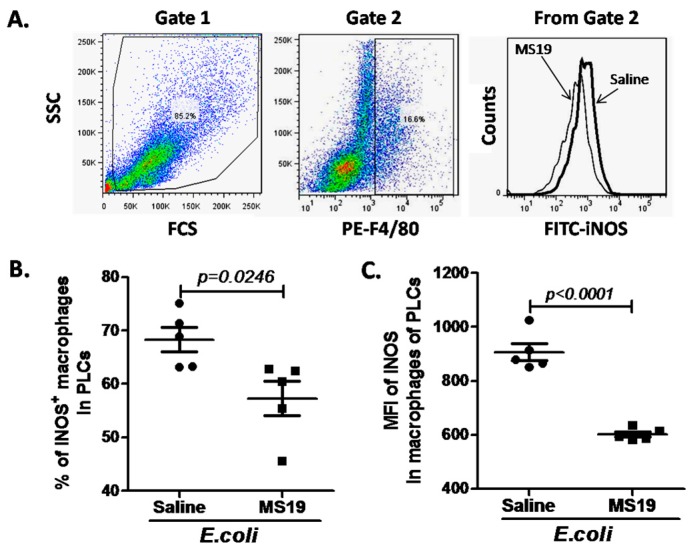
Down-regulatory effect of MS19 on the expression of inducible nitric oxide synthase (iNOS) in macrophages of PLCs from septic peritonitis model mice. The mice were intraperitoneally injected with *E. coli* at 1.6 × 10^8^ CFUs. At 1 h post infection, the mice were intraperitoneally administrated with (*n* = 5) or without (*n* = 5) MS19 (10 µg/mouse). At 8 h post-*E. coli* infection, the PLCs were harvested and then stained with PE-labeled anti-F4/80 mAb and fluorescein isothiocyanate (FITC)-labeled anti-iNOS mAb, followed by detection on a flow cytometry. (**A**) Gates for flow cytometry analysis; Left: Total of the PLCs. Middle: Ratios of iNOS^+^ macrophages. Right: Ratios of iNOS^+^ macrophages between the group of saline and MS19; (**B**) percentage of iNOS^+^ macrophages in PLCs; (**C**) mean fluorescence intensity (MFI) of iNOS in macrophages of PLCs. Each point represents the mRNA level from one mouse. Mean ± SD was marked with bars. The data were analyzed by SPSS 19.0 and followed the Gaussian distribution.

Considering that M1 macrophages are the classic or pro-inflammatory macrophages that produce TNF-α, IL-1, IL-6, and iNOS [9], and MS19 can reduce the mRNA levels of IL-6 and TNF-α, whether MS19 could inhibit the polarization of macrophages to M1 type by inhibiting the expression of iNOS, a prototypic marker of M1 macrophages, was investigated [21]. The PLCs were harvested from *E. coli*-infected mice with or without MS19 treatment at 8 h post-infection and stained with fluorescence-labeled anti-F4/80 and iNOS monoclonal antibody (mAb), followed by analyzing on a flow cytometry. After gating F4/80^+^ macrophages in PLCs, the iNOS^+^ macrophages were analyzed (Figure 3A). It was found that macrophages in the PLCs expressed increased level of iNOS, and MS19 could significantly reduce the ratios of iNOS^+^ macrophages (Figure 3B) and the iNOS expression levels on the macrophages (Figure 3C), indicating that MS19 could inhibit the polarization of macrophages to M1 type.

### 2.4. MS19 Can Down-Regulate the Production of IL-6 and TNF-α from Lipopolysaccharide (LPS)-Treated RAW264.7 Cells by Inhibiting Its Polarization to M1 Lineage

**Figure 4:**
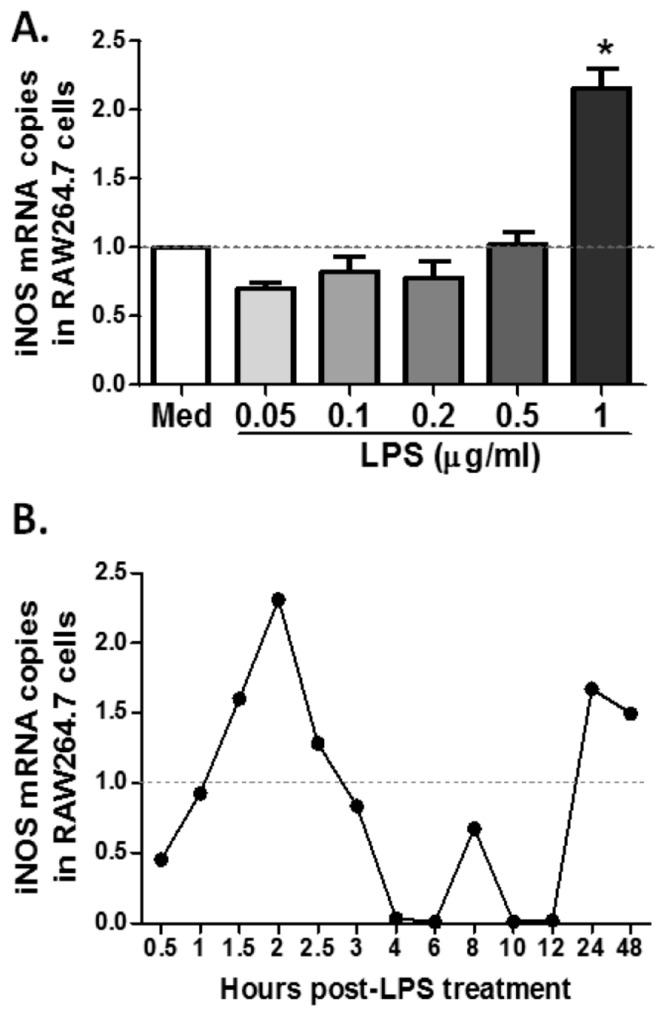
Effects of lipopolysaccharide (LPS) on iNOS production in RAW264.7 cells. RAW264.7 cells were plated in 6-well plates at a density of 1 × 10^6^ cells/well in Roswell Park Memorial Institude (RPMI) 1640 medium/10% FCS, treated with different dose of LPS for different times indicated in the figure, and then harvested for evaluating the mRNA levels of iNOS by qRT-PCR. The horizontal dotted line represents the normalized standard of glyceraldehyde-3-phosphate dehydrogenase (GAPDH) mRNA level. The data were the representative mean values of three independent experiments. (**A**) RAW264.7 cells were treated with LPS (0.05–1 µg/mL) for 2 h; (**B**) RAW264.7 cells were cultured with LPS at 1 µg/mL for 0.5 to 48 h.

**Figure 5:**
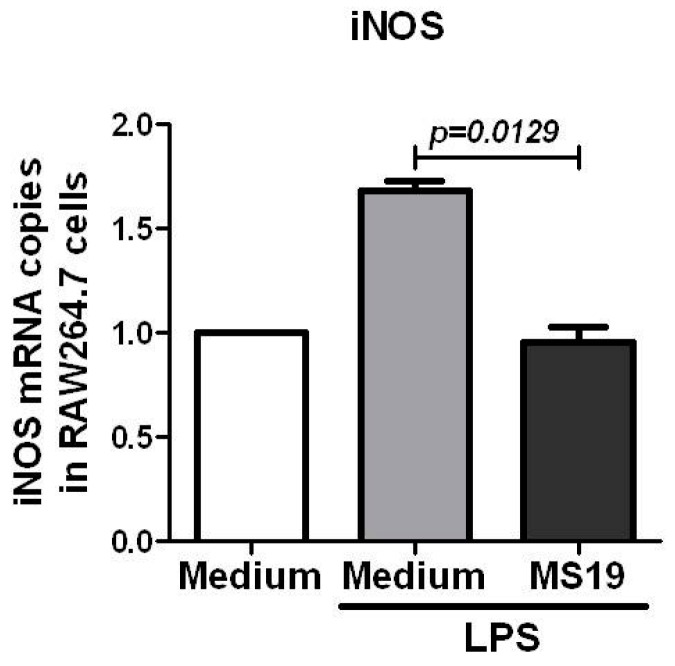
MS19 down-regulated the mRNA Levels of iNOS in LPS-treated RAW264.7 cells. RAW264.7 cells were treated with LPS (1 µg/mL) or LPS (1 µg/mL) + MS19 (8 µg/mL) for 2 h, and then lysed to isolate total RNA for amplifying mRNA by qRT-PCR. GAPDH mRNA level was as normalized standard. The data were representative of three independent experiments.

**Figure 6:**
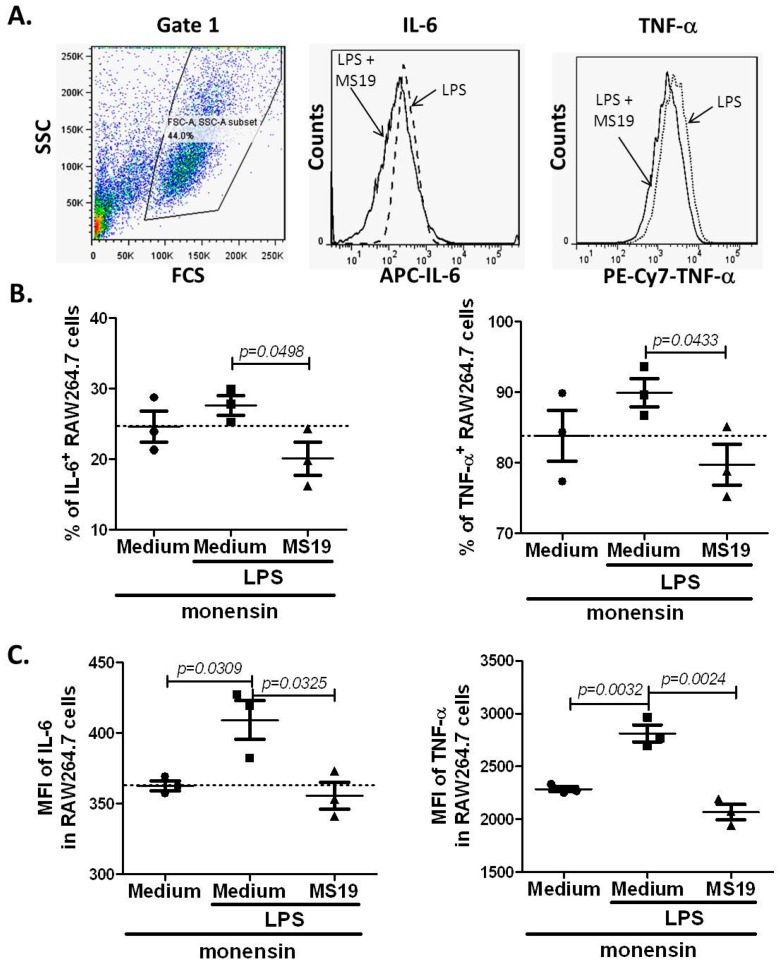
MS19 down-regulated the expression levels of IL-6 and TNF-α protein in LPS-treated RAW264.7 cells. RAW264.7 cells were cultured in medium containing LPS (1 µg/mL) + monensin (1 µL/mL) or LPS (1 µg/mL) + monensin (1 µL/mL) + MS19 (8 µg/mL) for 4 h, and then stained with APC rat anti-mouse IL-6 and PE-Cy7 rat anti-mouse TNF-α mAb. Afterward, the cells were analyzed on a flow cytometry. The data were representative of three independent experiments. (**A**) Gates and data from flow cytometry analysis. Left: Total of the RAW264.7 cells. Middle: Ratios of IL-6^+^ RAW264.7 cells between the group of saline and MS19. Right: Ratios of TNF-α^+^ RAW264.7 cells between the group of saline and MS19; (**B**) Percentage of IL-6^+^ and TNF-α^+^ in the RAW264.7 cells; (**C**) MFI of IL-6 and TNF-α in the RAW264.7 cells.

To further explore the mechanism by which MS19 can inhibit the polarization of macrophages to M1, this study tried to establish a cell model to simulate the septic peritonitis in mice by using RAW264.7 cells. The RAW264.7 cells, collected in American Type Culture Collection (ATCC), are monocyte/macrophage cell line cells established from tumor of male adult BALB/c mice induced by Abelson murine leukemia virus. The cells are typically used to study the macrophages (M1) differentiation [22]. For selecting the optimal dose of LPS, the RAW264.7 cells were cultured with LPS at dosages in a range of 0.05 to 1 µg/mL for 2 h. It was found that LPS at 1 µg/mL significantly elevated the mRNA level of iNOS (Figure 4A). Next, the effects of LPS at 1 µg/mL on mRNA expression of iNOS in RAW264.7 cells cultured from 0.5 to 48 h were kinetically observed. The result showed that iNOS mRNA reached the highest level in the RAW264.7 cells cultured with LPS for 2 h (Figure 4B). Upon these observations, 1 µg/mL of LPS and 2 h stimulation were selected to conduct the following in vitro experiments. To investigate whether MS19 could inhibit the expressions of iNOS, RAW264.7 cells were cultured with or without MS19 in the present of LPS for 2 h and then lysed to isolate total RNA for amplifying mRNA by quantitative reverse transcription-polymerase chain reaction (qRT-PCR). As shown in Figure 5, MS19 significantly reduced the mRNA levels of iNOS in the cells. Besides iNOS, the pro-inflammatory cytokines (IL-6 and TNF-α) were also produced by M1 macrophages [9]. Thus, the expressions of IL-6 and TNF-α were observed in the RAW264.7 cells. The cells were cultured with or without MS19 in the present of LPS and monensin. Monensin is a protein transport inhibitor which inhibits the secretion of IL-6 and TNF-α [23]. Four hours later, the cells were harvested and stained with allophycocyanin (APC) rat anti-mouse IL-6 and PE-Cy7 rat anti-mouse TNF-α mAb, followed by analyzing on a flow cytometry (Figure 6A). It was found that MS19 can obviously reduce the percentage of IL-6^+^ cells and TNF-α^+^ cells (Figure 6B), and protein expression of IL-6 and TNF-α in RAW264.7 cells (Figure 6C). Together, MS19 was demonstrated to inhibit the M1 polarity of LPS-treated RAW264.7 cells.

### 2.5. MS19 Inhibits the Polarization of Macrophages to M1 Type Possibly by Interfering Interferon Regulatory Factor 5 (IRF5)

**Figure 7:**
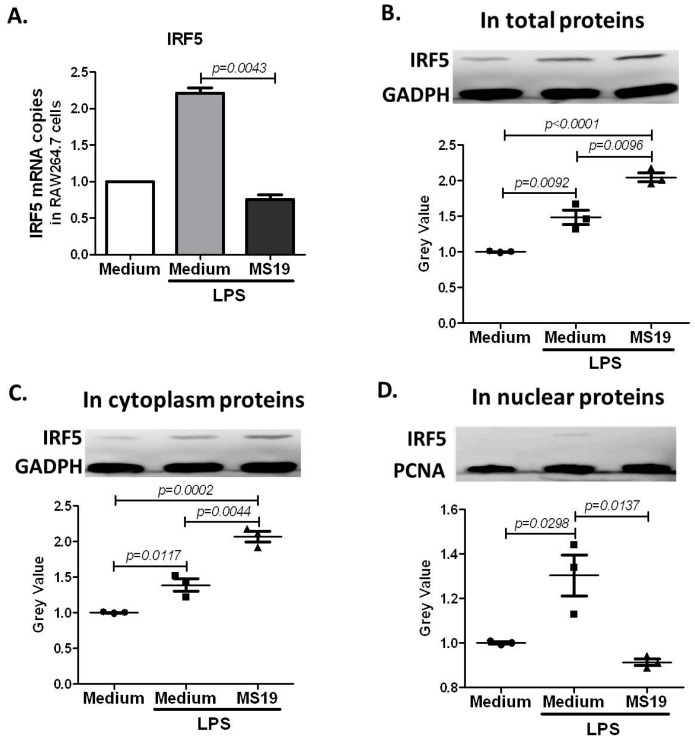
Effects of MS19 on the expression of IRF5 in LPS-treated RAW264.7 cells. RAW264.7 cells were cultured in medium with LPS (1 µg/mL) or LPS (1 µg/mL) + MS19 (8 µg/mL) for 2 h, and then analyzed for interferon regulatory factor 5 (IRF5) mRNA by qRT-PCR or protein by western blotting. GAPDH mRNA and protein were as normalized standards for IRF5 mRNA and protein in total or cytoplasm proteins, respectively. PCNA protein was as normalized standard for IRF5 protein in nuclear proteins. (**A**) IRF5 mRNA levels in total cell lysate. Data expressed as mean ± SD; (**B**) IRF5 protein levels in total proteins of the cells; (**C**) IRF5 protein levels in cytoplasm proteins of the cells; (**D**) IRF5 protein levels in nuclear proteins of the cells.

The transcription factor IRF5 is the major regulator of pro-inflammatory M1 macrophages polarization [13]. The binding motif in IRF5 is quite similar to the sequence of MS19. In order to prove whether MS19 inhibited M1 type polarization by interfering IRF5, the expression of IRF5 in the LPS-treated RAW264.7 cells was detected (Figure 7). As shown in Figure 7A, LPS increased the mRNA expression of IRF5 in the cells, while MS19 down-regulated the up-regulated IRF5 in the cells stimulated with LPS. Unexpectedly, an increased level of IRF5 proteins in the cells was found (Figure 7B). To find why IRF5 proteins were increased in the cells, accompanied with its down-regulated mRNA levels, the cytoplasm proteins and nuclear proteins of the cells were separated. After detection glyceraldehyde-3-phosphate dehydrogenase (GAPDH) and IRF5 in the plasma proteins, the proliferating cell nuclear antigen (PCNA) and IRF5 in the nuclear proteins of RAW264.7 cells by western blotting, we found that MS19 could significantly elevate cytoplasm IRF5 levels (Figure 7C) but reduce nuclear IRF5 levels (Figure 7D). These results suggested that MS19 inhibited IRF5 nuclear translocation from the cytosol, followed by interfering the initiation of its target genes.

## 3. Discussion

This study showed that MS19, an AAAG-rich ODN, displayed an immune regulatory role in interfering IRF5 on a septic peritonitis model mice infected by *E. coli*, and reduced the inflammatory response induced by elevated pro-inflammatory cytokines. To find the underling mechanisms, RAW264.7 cells were chosen to study how MS19 could influence the polarization of macrophages stimulated with LPS, and it was found that MS19 could inhibit the expression of iNOS and pro-inflammatory factors including IL-6 and TNF-α. This inhibition could be attributed to the interference of nuclear transportation of IRF5 by MS19, as demonstrated in the LPS-treated RAW264.7 cells (Figure 7). The interference could lead to the reduced activation of the genes of pro-inflammatory cytokines. The data suggested that MS19 had a direct effect on inhibiting the inflammatory M1 macrophages by interfering IRF5. The experimental studies using macrophage cell line like RAW264.7 cells could be translated into clinical benefits for the patients with sepsis because M1 macrophages contributed the development of the sepsis by producing great amount of pro-inflammatory cytokines [24]. Possibly, MS19 could be used as therapeutic oligonucleotides for the treatment of sepsis in humans by inhibiting IRF5 in the dysfunctional macrophages.

**Figure 8:**
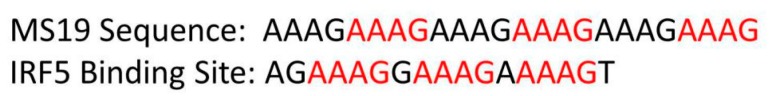
Sequences of MS19 and IRF5 binding site.

Being displayed inhibitory activity, MS19 could be a type of regulatory ODN (rODN). As established, the rODNs can be divided into four types according to their features of sequences and activities. Type I rODNs are guanine rich oligodeoxynucleotides, such as ODNs of 2114, 2088, and H154, and display inhibitory effect on CpG ODN-induced immune responses, such as proliferation of B cells, production of cytokines (e.g., IL-6, IL-12 and IFN-γ) by mouse spleen cells, and secretion of IFN-α by pDCs. Type II rODNs are oligodeoxynucleotides with TTAGGG repeat based on mammalian chromosome telomere sequences, such as A151, which is a typical rODN with broad immunosuppressive activities. Type III rODNs are oligodeoxynucleotides with tandem quinine sequences. Type IV rODNs are longer sulfur modified oligodeoxynucleotides and can compete TLR9 with CpG ODN in a length-dependent manner. Comparatively, MS19 is unique in sequence and activities. As shown in Figure 8, MS19 is consensus with the IRF5 binding site [25,26]. The AAAG repeats are existent in both. The sequence similarity can render MS19 to bind IRF5. The binding can interfere nuclear translocation of the IRF5, maintain the IRF5 protein in cytoplasm, and therefore decrease entry of the IRF5 into the nucleus. Thus, MS19 plays a role on inhibiting the production of pro-inflammatory cytokines from macrophages by interfering nuclear translocation of IRF5.

IRF5 has been demonstrated as a crucial regulator of the cell cycle, apoptosis [27,28], microbial infection [29,30] and inflammation [31,32]. IRF5 mediates growth inhibition by arresting G2-M cell cycle [27], sensitizes p53-proficient and p53-deficient colon cancer cells to DNA damage-induced apoptosis [28], and mediates inflammatory and immune responses by controlling expression of pro-inflammatory cytokines downstream of MyD88-dependent TLR signaling [32]. The current model of IRF5 activation includes the following stages: (1) Phosphorylation, ubiquitination, and possibly other post-translational modifications. Multiple amino acid residues have been suggested as potential IRF5 phosphorylation sites [29]; (2) Dimerization. Phosphorylation causes nuclear translocation of IRF5 by inducing a conformational change in the C-terminus of the protein, which enables dimerization and interaction with its co-factors [33]; (3) Nuclear transport. Once modified and dimerized, IRF5 is expected to go to the nucleus, as shown by LPS-induced IRF5 nuclear translocation in murine macrophages [34]; (4) Binding to gene promoters in complex with co-factors to regulate gene transcription.

## 4. Materials and Methods

### 4.1. Oligodeoxynucleotides

MS19 (5′-AAAGAAAGAAAGAAAGAAAGAAAG-3′) with full phosphorothioate modification was synthesized in Takara Co. (Dalian, China). It is diluted in PBS and tested for endotoxin by using the Limulus amebocyte lysate assay (Associates of Cape Cod, Inc., East Falmouth, MA, USA). All reagents used were pyrogen-free.

### 4.2. Cells

RAW264.7 cells (murine macrophage-like cell line) from ATCC, were obtained from Department of Molecular Biology, College of Basic Medical Sciences, Jilin University (Changchun, China). The cells were cultured at 37 °C in a 5% CO_2_ humidified incubator and maintained in RPMI 1640 medium (GIBCO, Shanghai, China) supplemented with 10% (*v*/*v*) heat-inactivated fetal bovine serum (FBS) and antibiotics (100 IU penicillin/mL and 100 IU streptomycin/mL).

### 4.3. Mice

Eight-week-old specific pathogen-free female ICR mice (20 ± 2 g) were obtained from the Experimental Animal Center, Jilin University (Changchun, China). The mice were maintained at 22 ± 2 °C with a 12 h light/dark cycle, and had free access to food and water for experiments in accordance with the National Institute of Health Guide for the Care and Use of Laboratory Animals.

### 4.4. Animal Experiments

The mice were housed for three days before manipulations. A well-established mouse model [19] was used to induce septic peritonitis. Briefly, the mouse model of *E. coli*-induced septic peritonitis was established by injecting with 1.6 × 10^8^ CFUs/mL intraperitoneally. To study the effect of MS ODN on the mortality, the model mice were injected intraperitoneally with MS19 (10 µg/mouse) at 1 h post infection. The survival of mice was recorded. The animal study was approved by the Ethics Committee of Jilin University (No. 2017-110, 1 January 2014).

### 4.5. Flow Cytometry of PLCs

For surface staining, the PLCs were stained with PE-conjugated anti-F4/80 monoclonal antibody for 30 min at room temperature in the dark followed by washing twice with PBS. For intracellular staining, the PLCs were surface stained as described above and then fixed with 4% paraformaldehyde and permeabilized with 0.1% saponin followed by staining with fluorescein isothiocyanate (FITC)-conjugated anti-iNOS monoclonal antibody.

In vitro experiment, the RAW264.7 cells were plated into 24-well plates at a density of 2 × 10^5^ cells/well, cultured with 1640 medium containing 2% FBS overnight. In next day, LPS (1 µg/mL), MS19 (8 µg/mL) and monensin (1 µL/mL, purchase from Abcom company, Cambridge, MA, USA) were added simultaneously. After four hours of incubation, cells were harvested and washed, fixed with 4% paraformaldehyde and permeablized with 0.1% saponin, followed by staining with APC rat anti-mouse IL-6 and PE-Cy7 rat anti-mouse TNF-α.

All stained cells were analyzed by flow cytometer Accuri C6 (BD) and FACSCanto (BD). Live cells were carefully gated by forward and side scattering. Data were analyzed with FlowJo software (FlowJo 7.6.1, LLC, Ashland, OR, USA).

### 4.6. RNA Isolation and Quantitative Reverse Transcription-Polymerase Chain Reaction (qRT-PCR)

Total RNA was extracted from cells using RNeasy Mini kit (Qiagen, Valencia, CA, USA) and reverse transcribed into cDNA using Taqman reverse transcription reagents (Applied Biosystems, Foster city, CA, USA) with random hexamer primer, according to the manufacturer’s instructions. The qRT-PCR was performed using SYBR Green and Light Cycler 480 system (Roche, Shanghai, China). The two-temperature cycle of 95 °C for 15 s and 60 °C for 1 min (repeated for 40 cycles) was used. Relative quantities of transcripts were calculated using the ΔΔCt method with GAPDH as a reference. The primers used for qRT-PCR were synthesized by Sangon Biotech (Shanghai, China) and listed in Table 1.

### 4.7. Western Blot Analysis

Whole cell extract and nuclear and cytoplasmic proteins were prepared from RAW264.7 cells by using a protein extraction kit (Beyotime, Shanghai, China) according to the manufacturer’s protocol. The protein concentration was measured by the Bicinchoninic acid (BCA) method. Samples (20 µg total proteins) were separated on 12% sodium dodecyl sulfate polyacrylamide gel electrophoresis (SDS-PAGE) and transferred to polyvinylidene fluoride (PVDF) membranes (Immobilon P; Millipore, MA, USA). After blocking with 5% nonfat milk in phosphate buffer solution with tween-20 (PBST) for 2 h at room temperature, the membranes were incubated overnight at 4 °C with primary antibody and then incubated with the conjugated secondary antibodies. The membranes were incubated with Electro-Chemi-Luminescence (ECL) detection kits for 1 min and then exposed to X-ray film. The intensity of the immunoreactive bands was determined using a densitometric analysis program (Image Gauge V3.12; Fuji Photo Film, Tokyo, Japan). Mouse monoclonal to IRF5, GAPDH and PCNA mAbs were purchased from Abcom company (Cambridge, MA, USA). Goat anti-mouse IgG was purchased from Zymed Laboratories (San Francisco, CA, USA).

### 4.8. Statistical Analysis

Statistical analysis was performed using SPSS 19.0 for Windows™. Comparisons between groups were conducted using analysis of Student’s *t*-tests. Survival curves of mice were estimated using the Kaplan–Meier method and compared using the log-rank test. Resulting *p* values of less than 0.05 were considered to be statistically significant.

## 5. Conclusions

Overall, the data provided here not only demonstrates the inhibitory roles of MS19 on macrophages involved inflammatory response in the development of sepsis, but also reveals a novel mechanism of MS19 mediated inhibition on IRF5 nuclear translocation in macrophages. Obviously, it is worthwhile to further probe the in vivo role of MS19 in development of sepsis and other inflammatory diseases.

## Figures and Tables

**Table 1:** Sequences of primers used in qRT-PCR.

Name	Sequence (5′–3′)
GAPDH	Forward: 5′-ATCACCATCTTCCAGGAGCGA-3′ Reverse: 5′-TCTCGTGGTTCACACCCATCA-3′
iNOS	Forward: 5′-CTGCTGGTGGTGACAAGCACATTT-3′ Reverse: 5′-ATGTCATGAGCAAAGGCGCAGAAC-3′
IRF5	Forward: 5′-AGCGGGAAGTCAAGACGAAGCTCT-3′ Reverse: 5′- CTGAGAACATCTCCAGCAGCA-3′
TNF-α	Forward: 5′-GGCTCCAGGCGGTGCTTGTT-3′ Reverse: 5′-GGCTTGTCACTCGGGGTTCG-3′
IL-6	Forward: 5′-GGATACCACTCCCAACAGACC-3′ Reverse: 5′-TCCAGTTTGGTAGCATCATCA-3′

GAPDH: *G*lyceraldehyde-3-phosphate dehydrogenase, iNOS: *I*nducible nitric oxide synthase, IRF5: *I*nterferon regulatory factor 5, TNF-α: *T*umor necrosis factor *α*, IL-6: interleukin-6.
